# Drainage network response to Arctic warming

**DOI:** 10.1038/s41467-023-40796-8

**Published:** 2023-09-12

**Authors:** Joel C. Rowland

**Affiliations:** Earth & Environmental Sciences Division, Los Alamos National Laboratory, Los Alamos, NM USA

**Keywords:** Hydrology, Environmental impact

## Abstract

Rapid Arctic warming may increase erosion and stream channel formation, which alters the flux of sediments, carbon, and nutrients in these sensitive ecosystems. Yet, understanding landscape change is hampered by a lack of predictive tools applicable to permafrost settings.

## Background

**Fig. 1:**
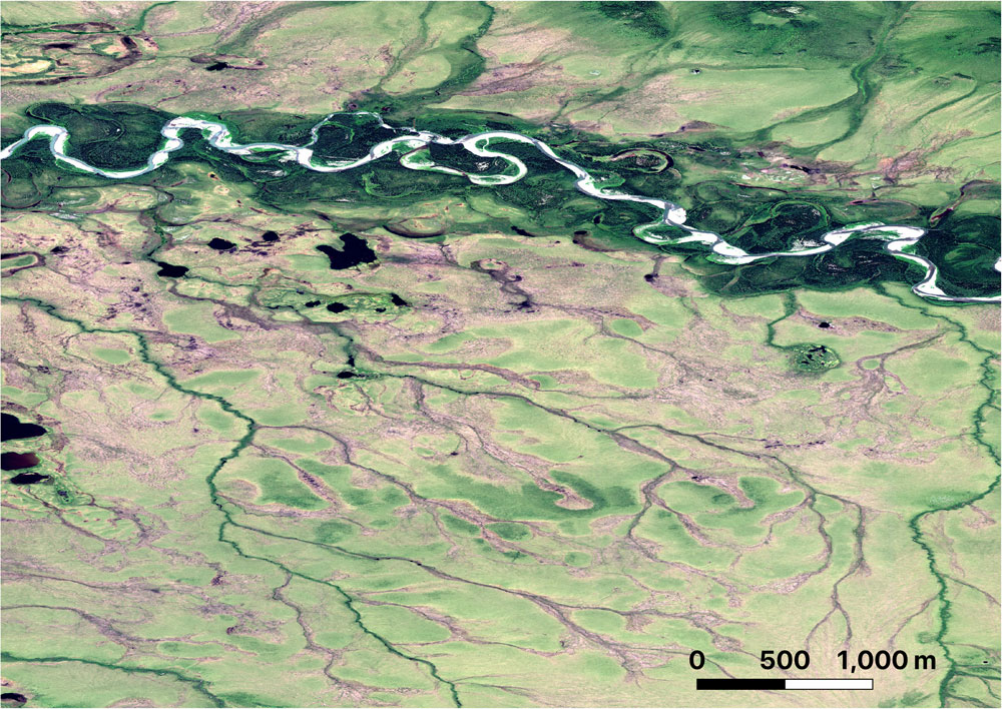
A Worldview2 satellite image of drainage networks at various states of development and connectivity along the south side of the Selawik River in northwest Alaska (66.463, −157.943). The channels are developing along networks of ice wedges and the orientations of the ice wedges remain imprinted on even the most developed channels (Maxar copyright 2011).

Thawing permafrost, melting ground ice, and changing hydrological regimes are all predicted to cause expansion of channel networks and increase hydrological connectivity across Arctic watersheds^1–4^ (Fig. 1). However, observed erosion of new channels have been isolated in both space and time and have yet to lead to widespread expansion new channelization or widespread evolution of Arctic watersheds. The presence of permafrost, ice in the ground, and the thermal sensitivity of land-surface processes in the Arctic has inhibited our ability to predict and quantify how a thawing Arctic landscape will alter fluxes of sediments, carbon, and nutrients into streams and rivers. Attempts to apply models developed in temperate landscapes for river channelization, landscape evolution and landslide occurrence have met with limited predictive success in permafrost landscapes^5–7^. In their study, Chartrand et al.^7^ address this challenge through reconstructing a 60-year history for landscape-scale channelization and evolution of the Muskox Valley (Axel Heiberg Island) in the high Arctic and demonstrate that factors typically used to predict channel development in non-permafrost regions do not control landscape evolution in the Arctic.

## Channel development and the role of ground ice

In non-permafrost watersheds, erosional gullies commonly develop in response to increases in erosive capability of surface water, decreases in the resisting forces of the land to erosion, or a combination of both. In these systems, gullying may be triggered by land use, climate change, or by natural cycles of erosion and deposition. In permafrost settings, gully formation by surface water flow commonly occurs by melting of ice wedges rather than erosion of the ground surface. Melting of ice wedges by flowing water creates tunnels that in turn collapse^8^. These collapse features remove protective vegetation and expose easily erodible soils to concentrated surface runoff leading to new channels with flow paths that closely follow the degrading ice wedge network^7,8^.

Documented in high-resolution topographic data, Chartrand et al.^7^ show that the development of channels within such settings is spatially discontinuous and independent of both topographic steepness of the valley and grain size of the channelized material, two common predictors for channel development in non-permafrost settings. Chartrand et al.^7^ further note the sources of water driving the melting of ice wedges are strongly influenced by thaw of permafrost and changing hydrology, such as snow melt magnitude and timing. All these factors make this type of channelization difficult to predict and require new models developed for these systems.

These challenges are not limited to settings with ice wedges. Subsurface erosion of soils due to water flowing through tunnels or pipes has also been observed in more southerly and discontinuous permafrost settings^9^, where ground ice may be more distributed and less predictable in location than the ice wedge complexes of Muskox Valley^7^. In these settings, subsurface flow also accelerates the melting of ground ice and physically erode soils from beneath the overlying, and often intact surface vegetation. Where pipes reconnect to the ground surface, large volumes of sediment and eroded organic matter may be deposited on top of undisturbed ground downslope^6^. Once sufficient volumes of ice melt and soil are excavated, the ground surface may collapse and drive channel development through coalescing disturbances^7^. Such channelization appears most commonly along valley axes where converging topography concentrates surface runoff.

## The potential role of landslides and water tracks in channel expansion

Rapid channelization along ice wedges is the most dramatic change highlighted in the Chartrand et al. study^7^, but they also document gullies, landslides, and linear surface water flow features called water tracks that all may play a role in expanding channel networks in permafrost watersheds. Along hillslopes across the Arctic, shallow landslides and deeper failures accelerate erosion and increase sediment and carbon delivery to streams and rivers^10,11^. Similar to ice wedge melt, these landslides tend to be isolated and generate local channel development rather than an integrated watershed-scale expansion of drainage networks. Attempts to predict the location and timing of these landslides with tools developed for temperate watersheds have met with limited success^5,6^. Results to date suggest that failures appear to be strongly connected to local ground ice conditions and seasonal warming, rather than topographic slope and hydrology^5^.

Figure 2 of the Chartrand et al. study^7^ shows numerous linear water track features. Water tracks are ubiquitous features in the Arctic that preferentially route water down hillslopes. The origin of water tracks is not well understood^12^ but it has been hypothesized that they may represent incipient drainage networks that will facilitate channel expansion in response to warming-induced erosion^13^. Erosion within water tracks have recently been observed and may be increasing^12,14^. However, a widespread integration of erosion along water tracks that would cause widespread channel expansion has not yet been documented. At present, the occurrence of water track erosion appears to be largely related to local ground collapse due to melting of ice^12^ rather than a systematic or predictable shift in processes governing channel development.

## Challenges to developing a predictive understanding of channelization in response to climate change

To address these research gaps, in their study of Muskox Valley, Canada, Chartrand et al.^7^ provide a watershed-scale picture of diverse processes that influence the flow of water and erosion. These processes range from failures on hillslopes to rapid erosion of ice wedge complexes along the valley axis. This study also highlights the complex interactions of ground ice, permafrost thaw, rapid warming, sediment grain sizes, and even stochastic flood events on channel formation. At present, we have very few tools for predicting where and how individual erosional processes occur in permafrost settings, let alone the ability to integrate these processes to predict channel network evolution across entire watersheds. The presence of ground ice strongly influences the vulnerability of permafrost landscapes to erosion and channel formation. Despite recent advances in the automated detection and mapping of ice wedge polygons^15^, we still lack the ability to map more distributed ground ice or predict which polygons may be vulnerable to surface water infiltration and melting.

More detailed watershed studies, such as the Muskox Valley study by Chartrand et al.^7^, that identify patterns and timing of channel expansion and link these changes to climatic and hydrological drivers, will be needed to develop mechanistic models of landscape evolution in permafrost settings. These models will need to incorporate thermal controls on landscape responses to warming and couple hillslopes to channels. Integrated models that capture hillslope and channel coupling will allow for predictions of how increased disturbance and erosion of permafrost soils will alter the storage, transport rates, and release pathways of sediment, carbon, and nutrients across watersheds in a rapidly warming Arctic.
